# Dieckol Ameliorates Aβ Production via PI3K/Akt/GSK-3β Regulated APP Processing in SweAPP N2a Cell

**DOI:** 10.3390/md19030152

**Published:** 2021-03-15

**Authors:** Jeong-Hyun Yoon, Nayoung Lee, Kumju Youn, Mi Ra Jo, Hyeung-Rak Kim, Dong-Seok Lee, Chi-Tang Ho, Mira Jun

**Affiliations:** 1 Department of Health Sciences, The graduate School of Dong-A University, Busan 49315, Korea; yjhyun1110@donga.ac.kr (J.-H.Y.); nylee0420@donga.ac.kr (N.L.); 2 Department of Food Science and Nutrition, Dong-A University, Busan 49315, Korea; kjyoun@dau.ac.kr; 3 Division of Food Safety and Processing Research, National Institute of Fisheries Science, Busan 46083, Korea; mirajo@korea.kr; 4 Department of Food Science and Nutrition, Pukyong National University, Busan 48513, Korea; hrkim@pknu.ac.kr; 5 School of Life Sciences & Biotechnology, College of Natural Sciences, Kyungpook National University, Daegu 41566, Korea; lee1@knu.ac.kr; 6 Department of Food Science, Rutgers University, New Brunswick, NJ 08901, USA; ctho@sebs.rutgers.edu

**Keywords:** Alzheimer’s disease, amyloid-beta peptide, GSK-3β, dieckol, SweAPP N2a

## Abstract

The proteolytic processing of amyloid precursor protein (APP) by β-secretase (BACE1) and γ-secretase releases amyloid-β peptide (Aβ), which deposits in amyloid plaques and contributes to the initial causative events of Alzheimer’s disease (AD). In the present study, the regulatory mechanism of APP processing of three phlorotannins was elucidated in Swedish mutant APP overexpressed N2a (SweAPP N2a) cells. Among the tested compounds, dieckol exhibited the highest inhibitory effect on both intra- and extracellular Aβ accumulation. In addition, dieckol regulated the APP processing enzymes, such as α-secretase (ADAM10), β-secretase, and γ-secretase, presenilin-1 (PS1), and their proteolytic products, sAPPα and sAPPβ, implying that the compound acts on both the amyloidogenic and non-amyloidogenic pathways. In addition, dieckol increased the phosphorylation of protein kinase B (Akt) at Ser473 and GSK-3β at Ser9, suggesting dieckol induced the activation of Akt, which phosphorylated GSK-3β. The specific phosphatidylinositol 3-kinase (PI3K) inhibitor LY294002 triggered GSK-3β activation and Aβ expression. In addition, co-treatment with LY294002 noticeably blocked the effect of dieckol on Aβ production, demonstrating that dieckol promoted the PI3K/Akt signaling pathway, which in turn inactivated GSK-3β, resulting in the reduction in Aβ levels.

## 1. Introduction

Alzheimer’s disease (AD) is a chronic neurodegenerative disorder accompanied with progressive memory loss and cognitive impairment. Amyloid-β (Aβ) peptides deposition is considered to be a primary hallmark in AD pathology. Insoluble Aβ is highly toxic and leads to synaptic dysfunction, glial cell activation and neuronal loss [1,2]. In addition, Aβ affects mitochondrial redox imbalance, causing oxidative stress and calcium overload, to induce cytokine release, and to disrupt acetylcholine and glutamate neurotransmission [3,4,5].

Aβ is a cleavage product of the amyloid-β precursor protein (APP) through the amyloidogenic pathway by β-site APP cleaving enzyme 1 (BACE1) and γ-secretase. BACE1 produces an extracellular APPβ (sAPPβ) fragment and a C-terminal fragment (C99). The C99 is subsequently cleaved by γ-secretase, leading to the production of Aβ peptides of various lengths (38–43 residues). Conversely, through a non-amyloidogenic pathway, APP is cleaved by α-secretase, mainly constituting a disintegrin and metalloproteinase domain-containing protein 10 (ADAM10), causing it to release soluble APPα (sAPPα), which thus precludes Aβ production. Li et al. reported that a slight increase in BACE1 expression is sufficient to promote Aβ formation under various pathological conditions [6]. In contrast, BACE1 gene deletion recovered Aβ-dependent memory deficits in a transgenic AD mice model, implying the importance of BACE as a rate-limiting enzyme in Aβ production [7]. An in vivo study by Ring et al. showed that sAPPα lowered Aβ generation and enhanced learning and neuronal plasticity in sAPPα knock-in mice [8], which suggests the positive physiological function of sAPPα.

The phosphoinositide 3-kinase (PI3K)/protein kinase B (Akt) pathway regulates various cellular functions, including survival, proliferation, migration and differentiation [9]. Several lines of evidence suggest that the PI3K/Akt signaling pathway plays pivotal roles in the central nervous system (CNS) via the regulation of neuronal survival. Once PI3K is activated by various stimuli, phosphoinositide-dependent protein kinase-1 (PDK1) allows the phosphorylation of Akt and activates it [10]. PI3K/Akt protects Aβ-induced toxicity, lowers tau hyperphosphorylation and restores memory impairment [11,12].

Glycogen synthase kinase 3β (GSK-3β) is one of the substrates of Akt [13]. GSK-3β is neurotoxic, and the phosphorylation of GSK-3β at serine 9 by activated Akt retains this enzyme in an inactive state. Importantly, GSK-3β, the principal isoform in the brain, is involved in neuropathological features such as Aβ deposition, tau protein hyperphosphorylation, synaptic dysfunction, and neuronal loss in AD [14,15,16]. A recent study suggested that the decreasing activity of GSK-3β obviously inhibited Aβ accumulation and alleviated memory dysfunction by a reduction in BACE1 expression in APP23/PS45 double transgenic mice. In addition, the knocking down of GSK-3β reduced BACE1 mRNA levels, but knocking down GSK-3α did not affect BACE1 expression [17]. Aβ exposure elevated GSK-3β activity via the PI3K pathway, while the suppression of either GSK-3β activity or its expression prevented Aβ-mediated neurotoxicity [18]. Recently, the activation of Akt inhibited GSK-3β by the phosphorylation of serine 9 residues during neuronal survival and glycogen synthesis, suggesting the PI3K/Akt pathway protected GSK-3β-evoked toxicity in neurons [19].

Marine organisms are a rich source of several natural molecules, such as terpenoids, polyphenols, polysaccharides, sterols, and peptides, which have various biologically effective properties, including antioxidative, antihypertensive, anti-obesity, antidiabetes, and anti-inflammatory properties [20,21,22,23]. Phlorotannins, polymers of phloroglucinol units, are found in brown algae such as *Sargassum* species, kelps and rockweeds. As a major phlorotannin, dieckol decreased the self-aggregation of Aβ_25–35_ with an IC_50_ value of 7.9 ± 0.2 [24]. Our group identified phlorotannins, including eckol, dieckol, and 8,8′-bieckol, as novel BACE1 inhibitors [25]. In addition, our previous study demonstrated that these phlorotannins suppressed Aβ-triggered apoptosis and inflammation via the NF-κB/MAPK signaling pathway in PC12 cell line [26]. However, the underlying mechanisms of phlorotannins in amyloidogenesis have not been clearly defined yet. Therefore, the present study demonstrated the effects and precise mechanisms of three phlorotannins on the proteolytic processing of APP in Swedish mutant APP overexpressed Neuro-2a cells (SweAPP N2a) for the first time.

## 2. Results and Discussion

### 2.1. Effects of Phlorotannins on Cell Viability

**Figure 1:**
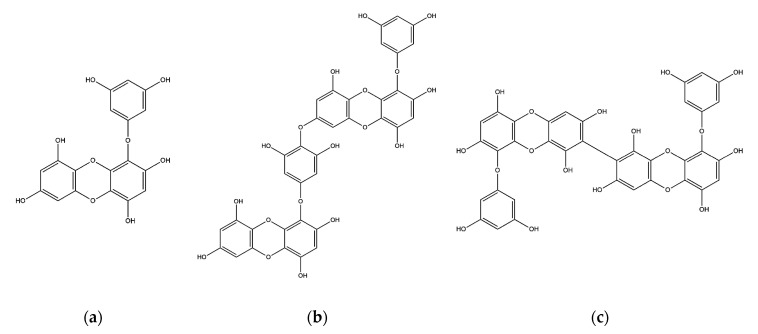
Structures of (**a**) eckol, (**b**) dieckol, and (**c**) 8,8′-bieckol.

**Figure 2:**
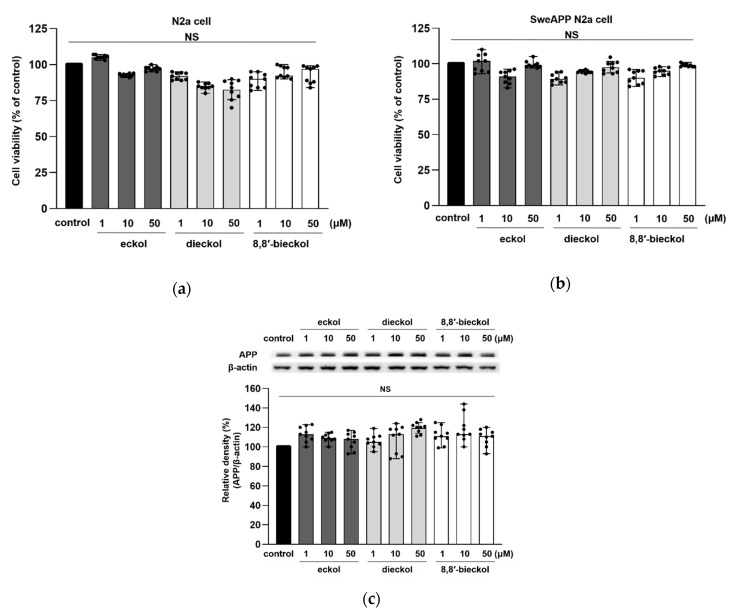
Effects of eckol, dieckol and 8,8′-bieckol on the cell viability. The viability of (**a**) N2a and (**b**) SweAPP N2a cells was measured using a 3-(4,5-dimethylthiazol-2-yl)-2,5-diphenyl-tetrazolium (MTT) assay. (**c**) APP protein levels in SweAPP N2a cells were detected by western blot. β-Actin was used as a normalizing control in the western blot assay. All phlorotannins showed no cytotoxicity in SweAPP N2a cell. The cells were treated with phlorotannins (1, 10, and 50 μM) for 24 h. The values are shown as the means ± SD from three independent experiments with 3 replications in each experiment. Black dots represent individual data point.

The chemical structures of eckol, dieckol, and 8,8′-bieckol are displayed in Figure 1. Three phlorotannins had no cytotoxic effects at 1, 10, and 50 μM in both N2a and SweAPP N2a cells (Figure 2a,b). In addition, there was no significant change in APP expression levels after the treatment of all the tested compounds (Figure 2c). Therefore, these concentrations were used in the following studies.

### 2.2. Inhibitory Effects of Phlorotannins on Aβ Production

**Figure 3:**
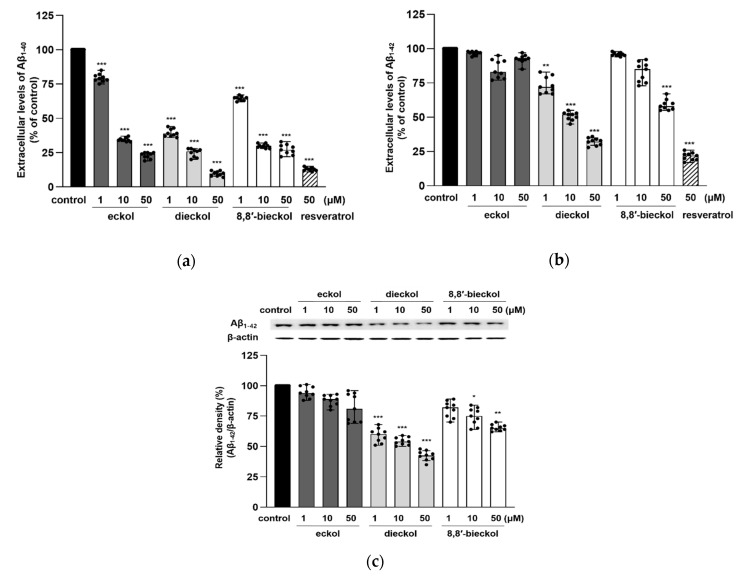
Effects of phlorotannins on the extracellular levels of (**a**) Aβ_1–40_ and (**b**) Aβ_1–42_ and (**c**) intracellular Aβ_1–42_ expression in SweAPP N2a cells. The SweAPP N2a cells were treated with phlorotannins (1, 10, and 50 μM) for 24 h, and the Aβ levels were measured with an Aβ ELISA kit and western blotting. β-Actin was used as a normalizing control in the western blot assay. The values are shown as the means ± SD from three independent experiments with 3 replications in each experiment. Black dots represent individual data point. *** *p* < 0.001, ** *p* < 0.01 and * *p* < 0.05 compared with control group.

Compared with N2a cells, SweAPP N2a cells secreted significantly larger amounts of extracellular Aβ_1–40_ and Aβ_1–42_ (data not shown). Dieckol and 8,8′-bieckol significantly decreased the extracellular level of Aβ species, Aβ_1–40_ and Aβ_1–42_, whereas eckol only, but dramatically, inhibited Aβ_1–40_ (Figure 3a,b). In particular, dieckol strongly attenuated Aβ_1–40_ production at all tested concentrations (*p* < 0.001), and noticeably regulated Aβ_1–40_ production similarly to resveratrol, which was used as a positive control. The results of the intracellular Aβ_1-42_ levels also showed that treatment with dieckol or 8,8′-bieckol significantly decreased the Aβ expression (Figure 3c). All tested compounds decreased Aβ production with no alteration in APP protein levels (Figure 3; Figure 2c).

Both extracellular and intracellular Aβ play critical roles in the onset of AD. It is commonly considered that extracellular Aβ is produced by the BACE1-mediated processing of APP and released in the extracellular matrix, which is a pivotal cause of AD [27]. However, recently, it has been shown that APP proteolysis can also occur in the endoplasmic reticulum, golgi apparatus and endosomes to produce intracellular Aβ. Moreover, Aβ can be taken up from extracellular sources through subsequent internalization [28,29,30]. In addition, according to previous studies, Aβ_1-42_ was considered to be the most neurotoxic Aβ species because it was more prone to aggregate and to initiate the formation of pathological oligomers, fibrils and plaques compared to the other forms [31,32,33]. Our present findings exhibited that hexamers of phloroglucinol, such as dieckol and 8,8′-bieckol, were more effective Aβ inhibitors than eckol, a trimeric compound of phloroglucinol.

### 2.3. Effects of Phlorotannins on APP Proteolytic Enzymes Expression and Activity

**Figure 4:**
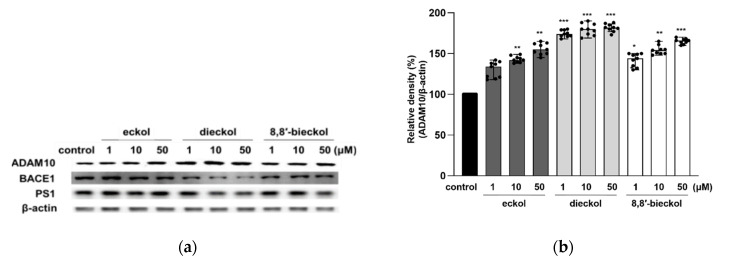

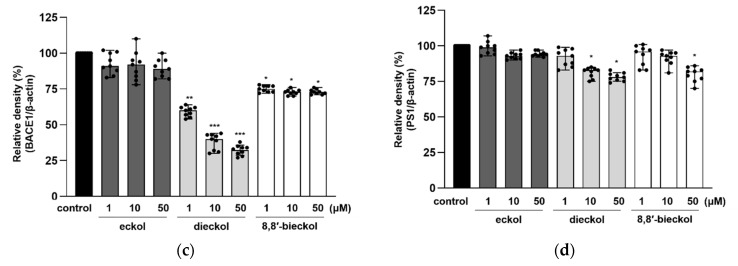
Effects of phlorotannins on levels of ADAM10, BACE1, and PS1 in SweAPP N2a cells. Western blotting was carried out to detect the expression of APP proteolytic enzymes. (**a**) Immunoblot bands and the quantifications of (**b**) ADAM10, (**c**) BACE1, and (**d**) PS1. The SweAPP N2a cells were incubated with phlorotannins (1, 10, and 50 μM) for 24 h. β-Actin was used as a normalizing control in the western blot assay. The values are shown as the means ± SD from 3 independent experiments with 3 replications in each experiment. Black dots represent individual data point. *** *p* < 0.001, ** *p* < 0.01 and * *p* < 0.05 compared with control group.

To confirm whether phlorotannins regulate the APP processing enzymes or not, protein expression of α-secretase (ADAM10), β-secretase (BACE1), γ-secretase and presenilin-1 (PS1) was evaluated. As shown in Figure 4a,b, the expression of ADAM10 was significantly increased by the pretreatment of all tested compounds. It is noteworthy that dieckol remarkably attenuated BACE1 expression even at the lowest concentration (Figure 4c). Additionally, 8,8′-bieckol significantly decreased BACE1 protein level (*p* < 0.05), while eckol showed no effect on BACE1. The results were consistent with our previous study showing the strong BACE1 inhibitory effect of dieckol with an IC_50_ value of 2.3 ± 0.1 in in vitro enzymatic system [25]. In the PS1 expression, shown in Figure 4d, statistically significant changes were detected as caused by dieckol at 10 and 50 μM (*p* < 0.05) and 8,8′-bieckol at 50 μM (*p* < 0.05).

**Figure 5:**
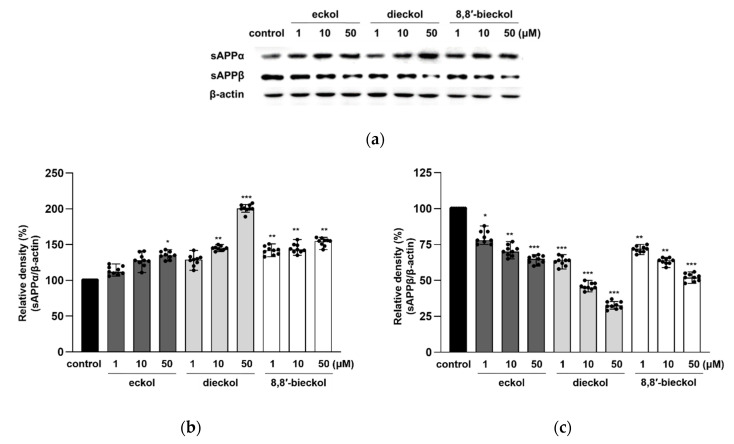
Effects of phlorotannins on sAPPα, and sAPPβ productions in SweAPP N2a cells. Western blotting was carried out to detect the expression of APP proteolytic products. (**a**) Immunoblot analysis and the quantification of (**b**) sAPPα and (**c**) sAPPβ. The SweAPP N2a cells were incubated with phlorotannins (1, 10, and 50 μM) for 24 h. β-Actin was used as a normalizing control in the western blot assay. The values are shown as the means ± SD from 3 independent experiments with 3 replications in each experiment. Black dots represent individual data point. *** *p* < 0.001, ** *p* < 0.01 and * *p* < 0.05 compared with control group.

For further investigation into the effects of the tested compounds on the activity of ADAM10 and BACE1, their proteolytic products, including sAPPα and sAPPβ, were measured. Consistent with the results of ADAM10 expression shown in Figure 4a,b, the protein levels of sAPPα were increased after dieckol and 8,8′-bieckol pretreatments (Figure 5). In particular, the expression level of sAPPα was dramatically elevated more than twofold after 50 μM dieckol pretreatment. The expression levels of sAPPβ were significantly reduced with the pre-treatment of all tested compounds.

It was demonstrated that ADAM10 and BACE1 compete for the same substrate, APP, and they have opposite effects on Aβ formation [34]. A previous study by May et al. (2011) revealed that the treatment of a non-peptidic BACE1 inhibitor, LY2811376, reduced sAPPβ and increased sAPPα in the human CNS, implying that the inhibition of BACE1 resulted in a compensatory augmentation in the cleavage of APP at the α-site [35]. Previously, a phlorotannin-rich extract from *Ecklonia cava* augmented the *C*-terminal fragment α (CTF-α) and sAPPα in SweAPP-HEK293 cells, which activity was similar to BACE inhibitor Ⅳ, suggesting that the extract acted as relative BACE inhibitor or TACE activator [36]. In a follow-up study by the same research group, the extract regulated the direct expression and substrate activity of α- and γ-secretase, leading to Aβ reduction [37]. These Aβ regulatory effects of the extract on APP processing were probably, in part, associated with our tested compound.

### 2.4. Regulation of Aβ Production by PI3K/Akt/GSK-3β Signaling Pathway

GSK-3β, highly expressed in the brain, has been identified as an in vivo substrate of the Akt pathway. The phosphorylation of GSK-3β via Akt is essential for the inhibition of GSK-3β during neuronal survival [38]. In addition, the regulation of GSK-3β by Akt is likely to affect other signaling events where GSK-3β is important, such as the hyperphosphorylation of tau [39].

**Figure 6:**
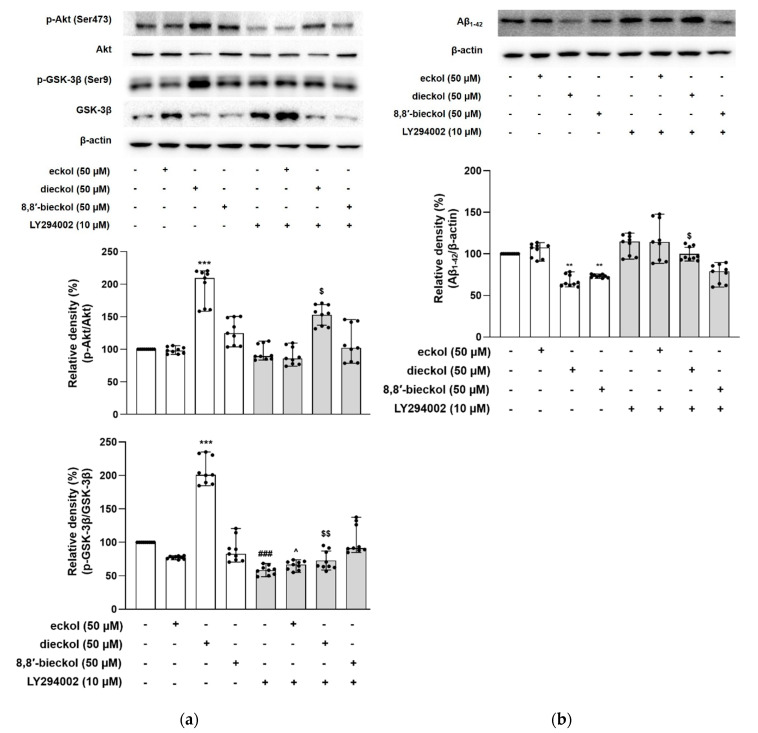
The regulatory effect of phlorotannins on PI3K/Akt/GSK-3β mediated Aβ production in SweAPP N2a cells. (**a**,**b**) LY294002 was used as PI3K inhibitor. (**a**) The expression levels of p-Akt (Ser473) and p-GSK-3β were determined by western blot analysis. The SweAPP N2a cells were pre-incubated with LY294002 for 30 min followed by stimulation with dieckol (50 µM) for 5 h. p-Akt and p-GSK-3β were normalized to total Akt and GSK-3β, respectively. (**b**) The expression levels of Aβ were determined by western blot analysis. The SweAPP N2a cells were pre-incubated with LY294002 for 30 min followed by stimulation with dieckol (50 µM) for 24 h. β-Actin was used as a normalizing control in the western blot assay. The values are shown as the means ± SD from 3 independent experiments with 3 replications in each experiment. Black dots represent individual data point. *** *p* < 0.001 and ** *p* < 0.01 compared with control group; ^###^ *p* < 0.001 compared with LY294002-untreated control group; ^^^ *p* < 0.05 compared with LY294002-untreated eckol group; ^$^ *p* < 0.05 and ^$$^ *p* < 0.01 compared with LY294002-untreated dieckol group. +, sample or LY294002 treated; -, sample or LY294002 untreated.

The GSK-3β signaling pathway is a critical regulator of APP processing, including BACE1 expression [17]. To determine whether the PI3K/Akt/GSK-3β signaling pathway could mediate the inhibitory effects of the tested phlorotannins on Aβ generation, phosphorylated levels of Akt at Ser473 and its downstream target GSK-3β at Ser9 were measured. As shown in Figure 6a, dieckol obviously increased the phosphorylation of Akt more than two-fold compared to the control group (*p* < 0.001). In addition, dieckol noticeably augmented the phosphorylation levels of GSK-3β at the Ser9 residue, subsequently inhibiting GSK-3β activity, suggesting the activated Akt downregulated GSK-3β activity (*p* < 0.001, Figure 6a). On the contrary, eckol or 8,8′-bieckol did not alter the phosphorylated levels of Akt and GSK-3β.

For further examination, the cells were exposed to PI3K inhibitor LY294002. LY294002 significantly decreased the level of phospho-GSK-3β compared to the control without LY294002. Interestingly, the combination of dieckol and LY294002 led to a markedly decreased phosphorylation of Akt and GSK-3β compared with the treatment with dieckol alone, suggesting that dieckol directly downregulated GSK-3β via the enhancement of PI3K/Akt.

Dieckol and 8,8′-bieckol significantly decreased the Aβ_1-42_ levels (67.33 ± 6.86% and 73.17 ± 0.54%, respectively) compared with the SweAPP N2a control group (*p* < 0.01, Figure 6b). However, the inhibitory effect of dieckol on Aβ was obviously blocked, when co-treated with LY294002, demonstrating that dieckol decreased Aβ via the PI3K/Akt-mediated inactivation of GSK-3β. These findings agree with a recent study exhibiting the reduction in Aβ_1-42_ levels upon GSK-3β inhibition in an APP/tau double transgenic mouse model [16]. To study the effects of GSK-3β reduction on Aβ formation, Ly et al. found that the reduced GSK-3β activity is involved in reducing the BACE1-mediated cleavage of APP and Aβ production by decreasing BACE1 gene transcription and expression in cell cultures and APP23/PS45 double transgenic mice [17]. In addition, the GSK-3β inhibition not only attenuated intracellular neurofibrillary tangles (NFTs), but also recovered memory deficits. These findings indicate that GSK-3β appears to be a common molecular connection in both amyloidogenesis and tau abnormalities.

Previous studies reported that Aβ production via APP processing was affected by several signaling pathways, such as PI3K/Akt/GSK-3β, extracellular signal-related kinase (ERK)/cAMP-response element binding protein (CREB)/peroxisome proliferator-activated receptor gamma (PPARγ), and NF-κB [40,41,42,43]. Ly et al. reported that GSK-3β elevates the transcriptional levels of BACE1 through NF-ĸB activation, thereby facilitating Aβ production [17]. Even though the exact mechanism of AD remains unclear, the strong anti-inflammatory property of dieckol via the NF-ĸB and MAPKs signaling pathway [26], together with the present results regulating the PI3K/Akt/GSK-3β signaling pathway, might partly mediate Aβ production.

Several recent studies on active compounds within marine organism and plants have paid attention to the modulation of the Akt/GSK-3β signaling pathway. Eckmaxol, one of the unique phlorotannins, significantly reversed the decreased expression of phosphorylated GSK-3β in Aβ-oligomer-evoked neurotoxicity. Eckmaxol revealed a favorable interaction in the ATP binding site of GSK-3β [44]. Fucoxanthin, a marine carotenoid, attenuated Aβ-oligomer-induced neurotoxicity via the PI3K/Akt/GSK-3β and ERK pathway [45]. Polypeptides from *Achyranthes bidentate* suppressed neuronal apoptosis via regulating the PI3K/Akt/GSK-3β pathway [46]. Curcumin attenuated cognitive function by inhibiting the hyperphosphorylated Tau protein through Akt/GSK-3β signaling [47]. Puerarin, a major isoflavone from *Pueraria lobata*, protected neuronal damage via the Akt/GSK-3β signaling pathway in cerebral ischemic animal models [48]. In addition, berberine, an alkaloid derived from *Berberis* species, was known to restore the Akt/GSK-3β pathway in primary cultured neuron and diabetic encephalopathy rats [49].

Neurons in the CNS are vulnerable to neurotoxic stimuli such as oxidative stress, microglia activation, inflammation, and glutamate toxicity. Dieckol and eckol exhibited potential protective efficacy against H_2_O_2_-stimulated neuronal death in murine hippocampus neuronal (HT22) cells [50]. Dieckol inhibited lipopolysaccharide (LPS)-induced microglial activation via the MAPK, Akt, and nicotinamide adenine dinucleotide phosphate (NADPH) oxidase-mediated pathways [51].

Glutamate is the major excitatory neurotransmitter in the CNS. Glutamatergic synaptic transmission has been implicated in learning and memory, and synaptic plasticity. However, high levels of extracellular glutamate lead to neuronal death under certain pathological conditions. In addition, Aβ promotes glutamatergic excitotoxicity and potently disrupts synapses and plasticity, providing an explanation for the cognitive deficits in AD [52]. Cui et al. reported that dieckol exerted neuroprotective effects in glutamate-induced mitochondrial-dependent apoptosis by activating the nuclear factor-like 2 (Nrf2)/heme oxygenase-1 (HO-1) pathway in both the primary cortical neurons and the HT22 neurons [53].

In terms of the structure–activity relationship, the numbers and positions of hydroxyl groups on the phenol ring are usually responsible for the neuroprotective properties of phenolic compounds. Dieckol (IC_50_, 2.2 µM) revealed a five-fold higher BACE1 inhibitory activity compared with that of eckol (IC_50_, 12.2 µM) [25]. Furthermore, the molecular size of phlorotannins is a vital factor for a strong interaction with the targeted enzymes, demonstrating that hexamers of phloroglucinol act as a better inhibitor [54]. The Aβ protective property of dieckol with a diphenyl ether linkage was greater than that of 8,8′-bieckol with a biaryl linkage, although these two compounds are dimers of eckol [26]. A recent study suggested that dieckol had a four-fold stronger inhibitory property against Aβ_25-35_ self-aggregation than eckol, which was achieved by interrupting the formation of the β-sheet-rich Aβ structures [24].

The present study revealed that dieckol effectively regulated the PI3K/Akt pathway, which in turn down-regulated GSK-3β activity, resulting in the suppression of Aβ production by modulation of the expression and activity of the APP processing enzymes. Although further in vivo study is definitely required, the present study itself is still meaningful in the terms of discovering potential anti-AD agents from marine organisms. As a next step towards expanding our research, neuronal primary cultures as well as animal studies will be performed.

## 3. Materials and Methods

### 3.1. Cell Culture

The N2a cells were supplied by the American Type Culture Collection (ATCC, Manassas, VA, USA). SweAPP N2a cells were kindly provided by Professor Dong-Seok Lee (Kyungpook National University, Daegu, Korea). The cells were maintained in 55% opti-minimal essential medium (MEM) (Gibco, Invitrogen Corporation, Burlington, Ontario, Canada) and 45% Dulbecco’s modified Eagle’s medium (DMEM) (Welgene, Daegu, Korea) with 10% fetal bovine serum (FBS) and 1% penicillin/streptomycin (Welgene, Daegu, Korea) in 5% CO_2_ at 37 °C (All from Hyclone Laboratories, Logan, UT, USA). Cells were selected with 500 ng/mL G418 (Sigma-Aldrich). Three phlorotannins, including eckol, dieckol, and 8,8′-bieckol, were generously provided by Professor Hyeung-Rak Kim (Pukyung National University, Busan, Korea). LY294002, a PI3K inhibitor, was purchased from Calbiochem (Darmstadt, Germany).

### 3.2. Cell Viability

SweAPP N2a cells and N2a cells were seeded in 96-well plates at a density of 5 × 10^5^ cells/mL treated with eckol, dieckol, and 8,8′-bieckol (1, 10, and 50 µM) for 24 h. The cells were incubated with 3-(4,5-dimethylthiazol-2-yl)-2,5-diphenyl-tetrazolium (MTT) solution (5 mg/mL in PBS) for 3 h at 37 °C (Sigma-Aldrich, St. Louis, MO, USA). After incubation, the solution was removed and DMSO was added to dissolve the formazan crystals. The optical absorbance at 570 nm was analyzed with a microplate reader (FLX800, BioTeK, Winooski, VT, USA).

### 3.3. Aβ ELISA Analysis

The SweAPP N2a cells were cultured in 6-well plates at a density of 3 × 10^6^ cells per well. Conditioned media from the phlorotannins-pretreated and -untreated cells were collected. The levels of Aβ_1–40_ and Aβ_1–42_ were quantified using an ELISA kit according to the manufacturer’s protocol (Invitrogen, Carlsbad, CA, USA). The absorbance was measured using a microplate reader at 450 nm. The concentrations of Aβ_1–40_ and Aβ_1–42_ were determined by comparison with their standard curves.

### 3.4. Western Blotting Assay

SweAPP N2a cells were plated in 6-well plates at the density of 3 × 10^6^ cells/well and treated with eckol, dieckol, and 8,8ʹ-bieckol (1, 10, and 50 µM) for various durations. The cells were washed with phosphate buffered saline (PBS) and then lysed in extraction buffer (Cell Signaling Technology Inc., Beverly, MA, USA) containing protease inhibitor cocktail for 1 h (Tech & Innovation, Chuncheon, Korea). The protein concentrations were quantified by the BCA method. Equal protein solutions (25 µg) were mixed with a loading buffer and heated at 95 ˚C for 5 min. The proteins were separated by 10% SDS-PAGE, then transferred to polyvinylidene fluoride (GE, Health Care Life Sciences, Piscataway, NJ, USA) and blocked in 5% skim milk for 2 h, at RT. The membranes were incubated with primary antibodies against APP (1:1000, Immuno-Biological Laboratories (IBL), Fujioka, Japan), ADAM10 (1:1000, Santa Cruz Biotech, Santa Cruz, CA) BACE1 (1:500, IBL), PS1 (1:1000, Cell Signaling), sAPP-α (1:500, IBL), sAPP-β (1:500, IBL), GSK-3β (1:2000, Santa Cruz Biotech), p-GSK-3β (1:2000, Cell Signaling), p-Akt (Ser473) (1:2000, Cell Signaling), Akt (1:2000, Cell Signaling), β-actin (1:1000, Santa Cruz Biotech) or PCNA (1:2000, Cell Signaling) overnight at 4 °C. These membranes were washed with Tris-buffered saline with Tween 20 (TBST) buffer and incubated with secondary antibodies, such as horseradish peroxidase (HRP)-conjugated anti-goat IgG, anti-mouse IgG or anti-rabbit IgG secondary antibody. The blots were visualized using an enhanced chemiluminescence (ECL) western blotting kit (Amersham Biosciences, Piscataway, NJ, USA) and Atto EZ-capture (Tokyo, Japan). β-Actin was probed as an internal control to confirm that an equal amount of protein was loaded in each lane. In order to avoid quantification and measurement bias, all images were obtained under the same setting conditions. Densitometric analysis and quantification of the protein bands were performed using the ImageJ software (NIH, Bethesda, MD, USA).

### 3.5. Statistical Analysis

All data were expressed as mean ± SD and were representative of the results obtained from three independent experiments. Statistical analyses were performed using SAS software (version 9.3, SAS Institute, Cary, NC, USA). One-way analysis of variance (ANOVA) with post hoc Tukey test was used to assess for the multiple comparisons. Graphs were constructed with the GraphPad Prism 9.0.2 software (GraphPad Software Inc., San Diego, CA, USA).

## 4. Conclusions

The present study is the first to demonstrate that dieckol regulated the APP proteolytic processing and Aβ production via the regulation of the PI3K/Akt/GSK-3β signaling pathway. Furthermore, the addition of LY294002 counteracts all the effects of dieckol, demonstrating that Akt/GSK-3β is the primary pathway mediating Aβ production in SweAPP N2a cells. The present findings supported a better understanding of the vital role of dieckol in preventing AD, and its potential to be used as a promising source of anti-AD agents.

## References

[1] Eimer W.A., Vassar R. (2013). Neuron Loss in the 5XFAD mouse model of Alzheimer’s Disease correlates with intraneuronal Aβ42 accumulation and caspase-3 activation. Mol. Neurodegener..

[2] Tu S., Okamoto S., Lipton S.A., Xu H. (2014). Oligomeric Aβ-induced synaptic dysfunction in Alzheimer’s disease. Mol. Neurodegener..

[3] Cabezas-Opazo F.A., Vergara-Pulgar K., Pérez M.J., Jara C., Osorio-Fuentealba C., Quintanilla R.A. (2015). Mitochondrial dysfunction contributes to the pathogenesis of Alzheimer’s disease. Oxid. Med. Cell Longev..

[4] Minter M.R., Taylor J.M., Crack P.J. (2016). The Contribution of neuroinflammation to amyloid toxicity in Alzheimer’s disease. J. Neurochem..

[5] Govoni S., Mura E., Racchi M., Lanni C., Grilli M., Zappettini S., Salamone A., Olivero G., Pittaluga A., Marchi M. (2014). Dangerous liaisons between beta-amyloid and cholinergic neurotransmission. Curr. Pharm. Des..

[6] Li Y., Zhou W., Tong Y., He G., Song W. (2006). Control of APP Processing and Aβ generation level by BACE1 enzymatic activity and transcription. FASEB J..

[7] Ohno M., Sametsky E.A., Younkin L.H., Oakley H., Younkin S.G., Citron M., Vassar R., Disterhoft J.F. (2004). BACE1 deficiency rescues memory deficits and cholinergic dysfunction in a mouse model of Alzheimer’s disease. Neuron.

[8] Ring S., Weyer S.W., Kilian S.B., Waldron E., Pietrzik C.U., Filippov M.A., Herms J., Buchholz C., Eckman C.B., Korte M. (2007). The secreted β-amyloid precursor protein ectodomain APPsα is sufficient to rescue the anatomical, behavioral and electrophysiological abnormalities of APP-deficient mice. J. Neurosci..

[9] Yang J., Chen L., Yang J., Ding J., Rong H., Dong W., Li X. (2012). High mobility group box-1 induces migration of vascular smooth muscle cells via TLR4-dependent PI3K/Akt pathway activation. Mol. Biol. Rep..

[10] Tang Y., Liu P., Tian Y., Xu Y., Ren F., Cui X., Fan J. (2015). Overexpression of ribonuclease inhibitor defines good prognosis and suppresses proliferation and metastasis in human colorectal cancer cells via PI3K/AKT pathway. Clin. Transl. Oncol..

[11] Martín D., Salinas M., López-Valdaliso R., Serrano E., Recuero M., Cuadrado A. (2001). Effect of the Alzheimer amyloid fragment Aβ(25–35) on Akt/PKB kinase and survival of PC12 cells. J. Neurochem..

[12] Liu S.J., Zhang A.H., Li H.L., Wang Q., Deng H.M., Netzer W.J., Xu H., Wang J.Z. (2003). Overactivation of glycogen synthase kinase-3 by inhibition of phosphoinositol-3 kinase and protein kinase C leads to hyperphosphorylation of tau and impairment of spatial memory. J. Neurochem..

[13] Beurel E., Grieco S.F., Jope R.S. (2015). Glycogen synthase kinase-3 (GSK3): Regulation, actions, and diseases. Pharmacol. Ther..

[14] Hurtado D.E., Molina-Porcel L., Carroll J.C., MacDonald C., Aboagye A.K., Trojanowski J.Q., Lee V.M. (2012). Selectively Silencing GSK-3 Isoforms reduces plaques and tangles in mouse models of Alzheimer’s disease. J. Neurosci..

[15] Cho J.H., Johnson G.V. (2004). Glycogen synthase kinase 3β induces caspase-cleaved tau aggregation in situ. J. Biol. Chem..

[16] DaRocha-Souto B., Coma M., Perez-Nievas B.G., Scotton T.C., Siao M., Sánchez-Ferrer P., Hashimoto T., Fan Z., Hudry E., Barroeta I. (2012). Activation of Glycogen synthase kinase-3 beta mediates β-amyloid induced neuritic damage in Alzheimer’s disease. Neurobiol. Dis..

[17] Ly P.T., Wu Y., Zou H., Wang R., Zhou W., Kinoshita A., Zhang M., Yang Y., Cai F., Woodgett J. (2013). Inhibition of GSK3β-mediated BACE1 expression reduces Alzheimer-associated phenotypes. J. Clin. Investig..

[18] Takashima A., Noguchi K., Michel G., Mercken M., Hoshi M., Ishiguro K., Imahori K. (1996). Exposure of rat hippocampal neurons to amyloid Β peptide (25–35) induces the inactivation of phosphatidyl inositol-3 kinase and the activation of tau protein kinase I/glycogen synthase kinase-3β. Neurosci. Lett..

[19] Reddy P.H. (2013). Amyloid beta-induced glycogen synthase kinase 3β phosphorylated VDAC1 in Alzheimer’s disease: Implications for synaptic dysfunction and neuronal damage. Biochim. Biophys. Acta.

[20] Kim A.R., Shin T.S., Lee M.S., Park J.Y., Park K.E., Yoon N.Y., Kim J.S., Choi J.S., Jang B.C., Byun D.S. (2009). Isolation and identification of phlorotannins from *Ecklonia Stolonifera* with antioxidant and anti-inflammatory properties. J. Agric. Food Chem..

[21] Kim E.K., Tang Y., Kim Y.S., Hwang J.W., Choi E.J., Lee J.H., Lee S.H., Jeon Y.J., Park P.J. (2015). First evidence that *Ecklonia Cava*-derived dieckol attenuates MCF-7 human breast carcinoma cell migration. Mar. Drugs.

[22] Kim H., Kong C.S., Lee J.I., Kim H., Baek S., Seo Y. (2013). Evaluation of inhibitory effect of phlorotannins from *Ecklonia Cava* on triglyceride accumulation in adipocyte. J. Agric. Food Chem..

[23] Kang M.C., Wijesinghe W.A.J.P., Lee S.H., Kang S.M., Ko S.C., Yang X., Kang N., Jeon B.T., Kim J., Lee D.H. (2013). Dieckol isolated from brown seaweed *Ecklonia Cava* attenuates type ІІ diabetes in db/db mouse model. Food Chem. Toxicol..

[24] Seong S.H., Paudel P., Jung H.A., Choi J.S. (2019). Identifying phlorofucofuroeckol-A as a dual inhibitor of amyloid-β25-35 self-aggregation and insulin glycation: Elucidation of the molecular mechanism of action. Mar. Drugs.

[25] Lee J., Jun M. (2018). Dual BACE1 and cholinesterase inhibitory effects of phlorotannins from *Ecklonia cava*-an in vitro and *in silico* study. Mar. Drugs.

[26] Lee S., Youn K., Kim D.H., Ahn M.R., Yoon E., Kim O.Y., Jun M. (2018). Anti-neuroinflammatory property of phlorotannins from *Ecklonia cava* on Aβ_25–35_-induced damage in PC12 cells. Mar. Drugs.

[27] Vassar R., Bennett B.D., Babu-Khan S., Kahn S., Mendiaz E.A., Denis P., Teplow D.B., Ross S., Amarante P., Loeloff R. (1999). Beta-secretase cleavage of Alzheimer’s amyloid precursor protein by the transmembrane aspartic protease BACE. Science.

[28] Jang J.K., Park K.J., Lee J.H., Ko K.Y., Kang S., Kim I.Y. (2017). Selenoprotein S is required for clearance of C99 through endoplasmic reticulum-associated degradation. Biochem. Biophys. Res. Commun..

[29] Toh W.H., Tan J.Z.A., Zulkefli K.L., Houghton F.J., Gleeson P.A. (2017). Amyloid precursor protein traffics from the golgi directly to early endosomes in an Arl5b-and AP4-dependent pathway. Traffic.

[30] Furusawa K., Takasugi T., Chiu Y.W., Hori Y., Tomita T., Fukuda M., Hisanaga S.I. (2019). CD2-Associated protein (CD2AP) overexpression accelerates amyloid precursor protein (APP) transfer from early endosomes to the lysosomal degradation pathway. J. Biol. Chem..

[31] Kuperstein I., Broersen K., Benilova I., Rozenski J., Jonckheere W., Debulpaep M., Vandersteen A., Segers-Nolten I., Van Der Werf K., Subramaniam V. (2010). Neurotoxicity of lzheimer’s disease Aβ peptides is induced by small changes in the Aβ42 to Aβ40 ratio. EMBO J..

[32] Pauwels K., Williams T.L., Morris K.L., Jonckheere W., Vandersteen A., Kelly G., Schymkowitz J., Rousseau F., Pastore A., Serpell L.C. (2012). Structural basis or increased toxicity of pathological aβ42:aβ40 ratios in Alzheimer Disease. J. Biol. Chem..

[33] Roche J., Shen Y., Lee J.H., Ying J., Bax A. (2016). Monomeric Aβ_1–40_ and Aβ_1–42_ peptides in solution adopt very similar ramachandran map distributions that closely resemble random coil. Biochemistry.

[34] Selkoe D.J., Schenk D. (2003). Alzheimer’s disease: Molecular understanding predicts amyloid-based therapeutics. Annu. Rev. Pharmacol. Toxicol..

[35] May P.C., Dean R.A., Lowe S.L., Martenyi F., Sheehan S.M., Boggs L.N., Monk S.A., Mathes B.M., Mergott D.J., Watson B.M. (2011). Robust central reduction of amyloid-β in humans with an orally available, non-peptidic β-secretase inhibitor. J. Neurosci..

[36] Kang I.J., Jeon Y.E., Yin X.F., Nam J.S., You S.G., Hong M.S., Jang B.G., Kim M. (2011). Butanol extract of *Ecklonia Cava* prevents production and aggregation of beta-amyloid and reduces beta-amyloid mediated neuronal death. Food Chem. Toxicol..

[37] Kang I.J., Jang B.G., In S., Choi B., Kim M., Kim M.J. (2013). Phlorotannin-Rich *Ecklonia Cava* Reduces the production of beta-amyloid by modulating alpha-and gamma-secretase expression and activity. Neurotoxicology.

[38] Chong Z.Z., Li F., Maiese K. (2005). Oxidative Stress in the Brain: Novel Cellular Targets that Govern Survival during Neurodegenerative Disease. Prog. Neurobiol..

[39] Tokutake T., Kasuga K., Yajima R., Sekine Y., Tezuka T., Nishizawa M., Ikeuchi T. (2012). Hyperphosphorylation of Tau Induced by Naturally Secreted Amyloid-β at Nanomolar Concentrations is Modulated by Insulin-Dependent Akt-GSK3β Signaling Pathway. J. Biol. Chem..

[40] Durairajan S.S.K., Liu L.-F., Lu J.-H., Chen L.-L., Yuan Q., Chung S.K., Huang L., Li X.-S., Huang J.-D., Li M. (2012). Berberine ameliorates β-amyloid pathology, gliosis, and cognitive impairment in an Alzheimer’s disease transgenic mouse model. Neurobiol. Aging.

[41] Wang Z., Huang X., Zhao P., Zhao L., Wang Z. (2018). Catalpol Inhibits amyloid-β generation through promoting α-cleavage of APP in Swedish mutant APP overexpressed N2a Cells. Front. Aging Neurosci..

[42] Liu F., Wang Y., Yan M., Zhang L., Pang T., Liao H. (2013). Glimepiride attenuates Abeta production via suppressing BACE1 activity in cortical neurons. Neurosci. Lett..

[43] Paris D., Ganey N.J., Laporte V., Patel N.S., Beaulieu-Abdelahad D., Bachmeier C., March A., Ait-Ghezala G., Mullan M.J. (2010). Reduction of β-amyloid pathology by celastrol in a transgenic mouse model of Alzheimer’s disease. J. Neuroinflamm..

[44] Wang J., Zheng J., Huang C., Zhao J., Lin J., Zhou X., Naman C.B., Wang N., Gerwick W.H., Wang Q. (2018). Eckmaxol, a phlorotannin extracted from *Ecklonia maxima*, produces anti-β-amyloid oligomer neuroprotective effects possibly via directly acting on glycogen synthase kinase 3β. ACS Chem. Neurosci..

[45] Lin J.J., Yu J., Zhao J.Y., Zhang K., Zheng J.C., Wang J.L., Huang C.H., Zhang J.R., Yan X.J., Gerwick W.H. (2017). Fucoxanthin, a Marine Carotenoid, Attenuates beta-Amyloid Oligomer-Induced Neurotoxicity Possibly via Regulating the PI3K/Akt and the ERK Pathways in SH-SY5Y Cells. Oxid. Med. Cell. Longev..

[46] Shen Y., Zhang Q., Gao X., Ding F. (2011). An active fraction of *Achyranthes Bidentata* Polypeptides prevents apoptosis induced by serum deprivation in SH-SY5Y cells through activation of PI3K/AKT/Gsk3β pathways. Neurochem. Res..

[47] Wang H., Sui H., Zheng Y., Jiang Y., Shi Y., Liang J., Zhao L. (2019). Curcumin-primed exosomes potently ameliorate cognitive function in AD mice by inhibiting hyperphosphorylation of the tau protein through the AKT/GSK-3β pathway. Nanoscale.

[48] Tao J., Cui Y., Duan Y., Zhang N., Wang C., Zhang F. (2017). Puerarin Attenuates locomotor and cognitive deficits as well as hippocampal neuronal injury through the PI3K/Akt1/GSK-3β signaling pathway in an *in vivo* model of cerebral ischemia. Oncotarget.

[49] Wang S., He B., Hang W., Wu N., Xia L., Wang X., Zhang Q., Zhou X., Feng Z., Chen Q. (2018). Berberine alleviates tau hyperphosphorylation and axonopathy-associated with diabetic encephalopathy via restoring PI3K/Akt/GSK3β pathway. J. Alzheimer’s Dis..

[50] Kang S., Cha S., Ko J., Kang M., Kim D., Heo S., Kim J., Heu M., Kim Y., Jung W. (2012). Neuroprotective effects of phlorotannins isolated from a brown alga, *Ecklonia cava*, against H_2_O_2_-induced oxidative stress in murine hippocampal HT22 cells. Environ. Toxicol. Pharmacol..

[51] Cui Y., Park J., Wu J., Lee J., Yang Y., Kang M., Jung S., Park J., Yoo E., Kim S. (2015). Dieckol Attenuates Microglia-mediated Neuronal Cell Death via ERK, Akt and NADPH Oxidase-mediated Pathways. Korean J. Physiol. Pharmacol..

[52] Parihar M.S., Brewer G.J. (2010). Amyloid-β as a modulator of synaptic plasticity. J. Alzheimer’s Dis..

[53] Cui Y., Amarsanaa K., Lee J.H., Rhim J.K., Kwon J.M., Kim S.H., Park J.M., Jung S.C., Eun S.Y. (2019). Neuroprotective mechanisms of dieckol against glutamate toxicity through reactive oxygen species scavenging and nuclear factor-like 2/heme oxygenase-1 pathway. Korean J. Physiol. Pharmacol..

[54] Jung H., Oh S., Choi J. (2010). Molecular docking studies of phlorotannins from *Eisenia bicyclis* with BACE1 inhibitory activity. Bioorg. Med. Chem. Lett..

